# A Randomized Clinical Trial Comparing Pure Hyaluronic Acid to the Hyaluronic Acid–Calcium Hydroxylapatite Combination for Temporal Volumization

**DOI:** 10.1093/asjof/ojaf076

**Published:** 2025-06-25

**Authors:** Marcelo Germani, Samantha Vitale, Adriana M Geroldo, Thiago Teixeira, Helem Haysahida, Danielle Dias, Pietra Roschel, Fernanda Lima, Ana C N Carnevali, Victor R M Munoz-Lora

## Abstract

**Background:**

Hyaluronic acid (HA) and calcium hydroxylapatite (CaHA) are commonly used for facial volumization, with HA offering hydration and reversibility, whereas CaHA provides structural support and collagen biostimulation. Recently, a combination of HA and CaHA has been proposed to enhance both immediate volumization and long-term tissue remodeling. However, its efficacy for volume retention remains unclear.

**Objectives:**

In this study, the authors aim to compare the volumetric retention and clinical outcomes of HA alone vs a manually prepared HA–CaHA mixture in the temporal region over a 90-day follow-up.

**Methods:**

A randomized controlled trial included 20 patients (40 hemifaces) treated with either 0.5 mL of HA alone (G1) or a 40% HA and 60% CaHA mixture (G2). Fillers were injected subcutaneously using a 22 G cannula with a fan technique. Volumetric changes were assessed through 3-dimensional stereophotogrammetry, and the Allergan Temple Scale (ATS) evaluated qualitative outcomes.

**Results:**

Both groups showed significant ATS score improvements over time (*P* < .001), but volumetric analysis demonstrated greater retention in G1 (0.231 ± 0.255 mL) compared with G2 (0.028 ± 0.135 mL, *P* = .016).

**Conclusions:**

The hypothesized synergy of the HA–CaHA combination was not observed. HA alone provided superior long-term volumization, suggesting that manual HA–CaHA mixing may alter filler performance. Further studies should explore premixed formulations and optimize treatment protocols.

**Level of Evidence: 2 (Therapeutic):**

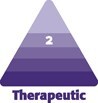

The high expectations of patients regarding aesthetic treatments often lead some professionals to choose procedures based solely on biological and physiological plausibility, despite lacking robust scientific evidence to support their efficacy and clinical safety.^1,2^ This practice, frequently driven by the desire to offer innovative treatments, can result in the adoption of methods that not only fail to meet the expected outcomes but also deprive patients of the opportunity to receive treatments that are evidence-based, scientifically validated, and considered the gold standard.^3^

One of the latest trends for facial volumization is to use a combination of hyaluronic acid (HA) with calcium hydroxylapatite (CaHA). This approach has gained popularity because of its apparent biological plausibility of integrating the benefits of both products into a single application: the volumizing effect of HA combined with the tissue quality improvement and high projection capacity of CaHA.^4-6^

Each HA formulation has distinct physicochemical properties that determine its indications, such as volumization (horizontal augmentation), structural support (vertical augmentation), hydration, and tissue repositioning.^7^ Additionally, HA-based products offer the advantage of being reversible, because they can be degraded by hyaluronidase in case of adverse events.^8,9^

On the other hand, CaHA formulations also vary significantly depending on their commercial preparation.^10^ Historically, CaHA was primarily used as a volumizer, similar to HA.^11^ However, its current use is predominantly focused on collagen biostimulation, expanding its clinical applications.^12^ Despite being nonreversible, CaHA is still applied in high-risk areas such as the temporal and frontal regions.^13^ Moreover, because of CaHA's low reversibility but potential for positive outcomes, some authors have suggested its combination with HA to enhance product safety. However, questions remain regarding whether this mixture offers superior volumization compared with HA alone.^14^

The temporal region plays a crucial role in facial aesthetics, contributing to overall harmony and balance.^15^ Age-related volume loss in this area, often referred to as temporal hollowing, is a common concern among patients seeking facial rejuvenation.^16^ This condition results from the progressive atrophy of fat compartments, bone resorption, and skin laxity, leading to a gaunt and aged appearance.^17^ The aesthetic impact of temporal hollowing extends beyond localized volume loss, because it influences the perception of youthfulness, facial proportions, and even gender characteristics.^18^ In clinical practice, the correction of temporal volume loss is increasingly sought after because of the growing demand for minimally invasive procedures that provide natural and long-lasting results.^19^

The authors of this study aim to objectively compare the volumizing effect of pure HA and a manually mixed combination of HA and CaHA in the treatment of temporal hollowing. By evaluating the volumetric retention and clinical outcomes of both approaches over time, this research seeks to determine whether the combination offers any advantages compared with HA alone in providing sustained augmentation for patients with temporal volume loss.

## METHODS

### Study Design

This randomized controlled and comparative clinical trial received ethical approval from the Kaiser Hospital Research Ethics Committee under protocol number CAAE—75193923.8.0000.0281. All participants provided written informed consent before enrollment. In this study, the authors’ followed Good Clinical Practice guidelines and adhered to the Declaration of Helsinki for research involving human participants. The present study is registered under the trial number U1111-1318-8337, ensuring compliance with ethical standards and providing transparency in the research process.

### Study Participants

Twenty volunteers of both sexes, aged 25 to 60 years, resulting in the evaluation of 40 treated temporal regions, seeking improvement in temporal hollowing, were recruited from a private clinic in Lins, São Paulo, Brazil, between April 1 and April 10, 2024. Participants were excluded if they had known allergies to any of the products used, had undergone any aesthetic treatment in the temporal region within the past 12 months, were pregnant or breastfeeding, had coagulation disorders, or declined to sign the informed consent form.

Volunteers were randomly allocated into 2 groups (*n* = 10) using a Random Allocation Software 2.0^20^:

Group 1 (G1): Participants received 0.5 mL of HA (Belotero Volume, Merz, Raleigh, NC) per hemiface.

Group 2 (G2): Participants received a mixture containing 40% HA (Belotero Volume) and 60% CaHA (Radiesse, Merz, Raleigh, NC) per hemiface. The mixture was prepared by transferring 0.4 mL of HA and 0.6 mL of CaHA into a 5.0 mL sterile syringe using a Luer-lock to Luer-lock connector (Descarpack, Ilhota, Santa Catarina, Brazil). The mixture was prepared manually by transferring the products between 2 syringes 30 times through a Luer-lock connector to achieve a visually homogeneous blend. No formal microscopic homogeneity testing was performed.

### Injection Technique

Before the procedure, all participants underwent skin asepsis, including the removal of any makeup or contaminants, followed by disinfection with a 2% chlorhexidine alcohol solution (Riohex, Rioquímica, São Paulo, Brazil). Local infiltrative anesthesia was administered at the entry point using 3% mepivacaine without vasoconstrictor (Mepisv, DFL, Rio de Janeiro, Brazil) to enhance patient comfort.

**Figure 1.**
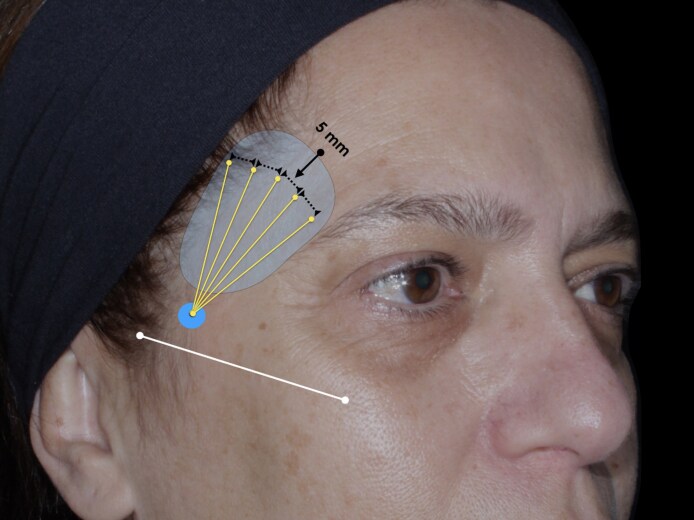
Schematic representation of the predefined treated and assessed temporal region. Blue circle represents the cannula entry point located 10 mm above the zygomatic arch (white line). Product was subcutaneously distributed along the delimited area using a fan technique (yellow lines) which each retroinjection separated by 5 mm each other.

All injections (regardless of the group) were performed by a single experienced practitioner with over 10 years of expertise in facial aesthetic procedures. A 24 G needle (Terumo, São Paulo, Brazil) was used to create the entry point, positioned just below the temporal fossa at the level of the zygomatic arch. The product was then administered into the subcutaneous plane of the temporal fossa, based on anatomical studies indicating lower vascular density compared with deeper planes in the temporal region, thereby optimizing safety and moldability for volumization purposes. The temporal fossa was defined superiorly at the inferior border of the superior temporal line; inferiorly, the region extended up to 10 mm above the zygomatic arch; anteriorly, the limit was demarcated by the posterior edge of the frontal bone near the lateral orbital rim; and posteriorly, the boundary was defined by a vertical line positioned 2 cm anterior to the tragus (Figure 1). A 22 G cannula (Biometik, Santa Catarina, Brazil) was employed, and the product was distributed following a fan technique, with 5 linear retrograde injections spaced 0.5 cm apart and directed toward the temporal fossa. Each line received 0.1 mL of the product (either HA alone or the mixture), totaling 0.5 mL per hemiface (Figure 1).

### Allergan Temple Hollowing Scale (Allergan Temple Scale)

**Figure 2.**
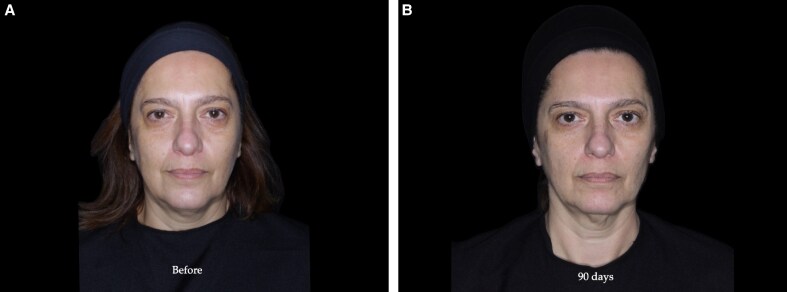
(A) Before and (B) 90 day assessment of a 48-year-old female patient treated with 1 mL of hyaluronic acid in the temporal region (0.5 mL on each side). The Allergan Temple Scale score was 3 bilaterally at baseline and improved to 1 on both sides at Day 90. Volumetric analysis showed a change of 0.20 mL on the right side and 0.35 mL on the left.

**Figure 3.**
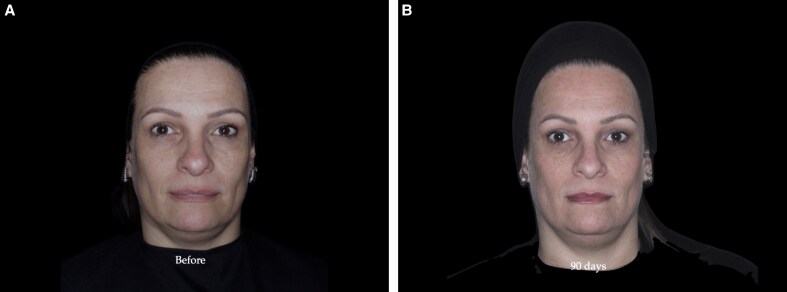
(A) Before and (B) 90 day assessment of a 46-year-old female patient treated with 1 mL of mixture containing 40% hyaluronic acid and 60% calcium hydroxyapatite in the temporal region (0.5 mL on each side). The Allergan Temple Scale score was 3 bilaterally at baseline and improved to 1 on both sides at Day 90. Volumetric analysis showed a change of 0.43 mL on the right side and 0.29 mL on the left.

The Allergan Temple Hollowing Scale (ATS) is a photo-numeric scale designed to assess the severity of volume loss in the temporal region.^21^ The scale classifies volume deficiency severity into 5 grades, ranging from 0 (normal convexity) to 4 (severe hollowing), with verbal descriptors and illustrative images assigned to each level (Table 1). Participants were evaluated using this scale at baseline and at 90 days posttreatment through in-person assessments performed by a single blinded evaluator with clinical experience in facial aesthetics, ensuring objective and consistent evaluation (Figures 2, 3).

**Table 1.** Allergan Temple Hollowing Scale^16^

Grade	Term	Characteristics
0	Convex	Rounded temple
1	Flat	Flat temple, temporal fusion line may be visible
2	Minimal	Shallow depression or concavity with minimal volume loss, temporal fusion line may be visible
3	Moderate	Moderate depression or concavity with moderate volume loss, moderate prominence of temporal fusion line
4	Severe	Deeply recessed, sunken appearance, marked prominence of temporal fusion line and zygomatic arch

### Volumetric Analysis

Volumetric analysis of the temple region was conducted by obtaining 3-dimensional (3D) images using a 3D stereophotogrammetry device (QuantifiCare, Sophia Antipolis, France). After photographic acquisition, the DermaPix Database software (QuantifiCare, Sophia Antipolis, France) automatically integrated stereo images to generate 3D facial reconstructions, which were processed and managed using the LifeViz application (QuantifiCare, Sophia Antipolis, France). This software enabled the calculation and visualization of volumetric differences between comparative images. All photographic acquisitions and analyses were performed by independent operators blinded to the study procedures, ensuring objective assessments.

Volumetric analyses were assessed in the anterior temporal region (as delimited above; Figure 1) and conducted at 3 time points: T0 (before treatment), T1 (immediately post treatment), and T2 (90 days post treatment). Volume changes were calculated by the difference between the posttreatment evaluations and baseline (T1-T0 and T2-T0). All stereophotogrammetry analyses were performed by a single trained operator following a standardized acquisition and processing protocol. The operator was blinded to group allocation to minimize assessment bias.

### Statistical Analysis

All statistical analyses were conducted using Jamovi statistical software (The Jamovi Project, version 2.3.28, Sydney, Australia), with a significance level of *P* < .05 for all tests. All data are presented as mean ± standard deviation to ensure clarity and consistency in reporting.

To compare baseline characteristics between the 2 groups (age, BMI, and ATS scores), independent samples *t*-tests were performed. For the evaluation of ATS scores and volumetric changes over time, repeated measures analysis of variance (ANOVA) was employed. Post hoc comparisons were conducted using Tukey's test when significant interactions or main effects were detected. These statistical methods allowed for the assessment of both within-group changes over time and between-group differences, ensuring a comprehensive evaluation of the data.

## RESULTS

### Demographics

The study included a total of 20 female patients, accounting for 40 temporal regions, with a mean age of 47.5 ± 6.98 years, and a mean BMI of 27.5 ± 4.40 kg/m^2^. When analyzing the groups separately, the mean age was 49.3 ± 7.91 years in G1 and 45.7 ± 5.53 years in G2, with no statistically significant difference between them (*P* = .103). The BMI was 26.4 ± 2.33 kg/m^2^ in G1 and 28.6 ± 5.65 kg/m^2^ in G2, also showing no significant difference (*P* = .126). All participants completed the study protocol without dropouts or missing data during the follow-up assessments.

### Temporal Depth (Allergan Temple Scale)

At baseline, G1 presented an ATS score of 2.40 ± 0.681 and G2 presented 2.40 ± 0.503, with no significant difference between them (*P* = 1.000).

Changes in temporal depth over time were evaluated using repeated measures ANOVA. A significant effect of time was observed with a mean difference score of −1.00 for G1 and −0.70 for G2 between baseline (Day 0) and 90 days (*P* < .001 for both groups), indicating that temporal depth scores decreased significantly over time across all groups.

**Figure 4.**
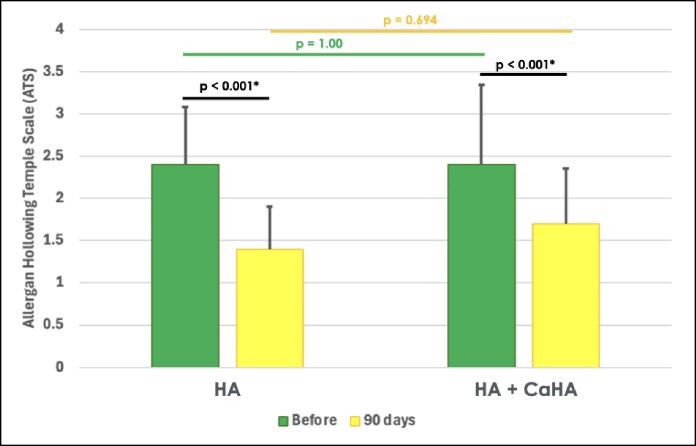
Bar graphs showing the mean and standard deviation (SD) of the Allergan Hollowing Temple Scale scores before and 90 days post treatment with hyaluronic acid (HA) or a mixture containing 40% HA and 60% calcium hydroxyapatite (HA + CaHA) in the temporal region. *P*-values indicate across group differences according to repeated measures analysis of variance followed by Tukey’s post hoc. Statistically significant *P*-values are marked with an asterisk.

However, there were no significant differences among groups at 90 days (*P* = .694), indicating that neither treatment demonstrated a superior improvement on temporal depth (Figure 4).

### Temporal Volumetric Changes

Volume changes at the temporal region were assessed using repeated measures ANOVA. No significant main effect of time was observed with *P* = .172 for G1 and *P* = .659 for G2, but a significant interaction between time and group was found (*P* = .027; effect size = 0.122), indicating that the pattern of volumetric change differed between groups over time. A significant main effect of group was also detected (*P* = .021; effect size = 0.132), suggesting that 1 treatment resulted in greater overall volume retention.

**Figure 5.**
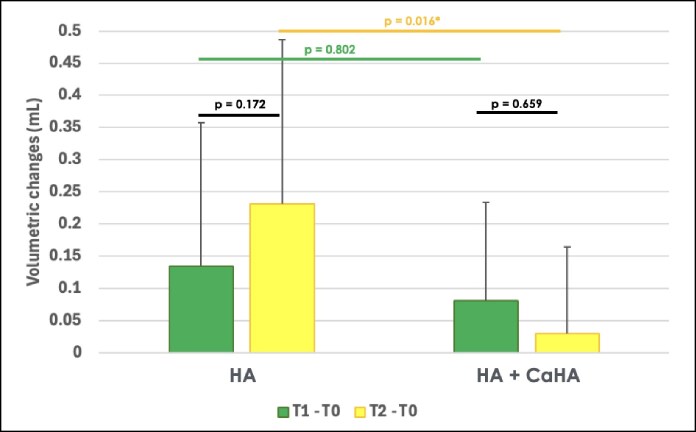
Bar graphs showing the mean and standard deviation (SD) of the volumetric changes (assessed by stereophotogrammetry) immediately after (T1-T0) and 90 days post treatment (T2-T0) with hyaluronic acid (HA) or a mixture containing 40% HA and 60% calcium hydroxyapatite (HA + CaHA) in the temporal region. *P*-values indicate across group differences according to repeated measures analysis of variance followed by Tukey’s post hoc. Statistically significant *P*-values are marked with an asterisk.

At the initial posttreatment assessment (T1-T0), both groups showed a similar volumetric increase, with G1 presenting a mean change of 0.135 ± 0.222 mL and G2 0.081 ± 0.152 mL, with no significant difference between them (*P* = .802). However, at the 90-day follow-up (T2-T0), G1 maintained a greater volume increase (0.231 ± 0.255 mL), whereas G2 showed almost no volumetric gain (0.028 ± 0.135 mL), resulting in a significant difference between groups (mean difference of 0.203 ± 0.064 mL, *P* = .016; Figure 5).

The effect size for the volumetric retention analysis was *η*^2^ = 0.122 for the time × group interaction and *η*^2^ = 0.132 for the between-group comparison, indicating moderate-to-large effects.

### Adverse Events

No serious adverse events were recorded during the study period. Minor local reactions, including mild edema and transient ecchymosis at the injection sites, were observed in a small number of patients in both groups. All minor events resolved spontaneously within a few days without the need for medical intervention. No differences in adverse event profiles were observed between the HA and HA–CaHA groups.

## DISCUSSION

Our results demonstrate the efficacy of both treatments in the qualitative assessment using the ATS, because both groups showed a significant reduction in temporal hollowing over time (*P* < .001). Although G1 (HA alone) presented more favorable outcomes, this difference was not statistically significant between the groups. In the quantitative analysis, however, G1 proved to be more effective in volumetric retention, demonstrating superior volume maintenance compared with G2 (HA + CaHA mixture). Although both treatments initially provided volumization, HA alone resulted in more sustained volumetric enhancement over time, suggesting that it may offer longer-lasting volumization effects.

Although the HA used in this study has a high elastic modulus, meaning it provides significant firmness, CaHA demonstrates even greater resistance to deformation.^6^ From a rheological standpoint—and consequently from a clinical perspective—1 might hypothesize that combining these 2 materials could result in a product with a higher firmness, potentially enhancing its tissue projection capabilities. However, our findings did not support this hypothesis because no differences were found at immediately posttreatment volume assessment.^10^

According to McCarthy et al, the dilution of CaHA significantly impacts its rheological properties, particularly its tissue projection capacity. They showed that because CaHA was diluted in increasing proportions, its elastic modulus (*G*′) decreased drastically, with reductions of up to 98% at a 1:1 dilution ratio. This implies that CaHA ability to provide direct filling and structural support is substantially compromised as dilution increases. This effect arises from alterations in the product's cohesiveness, making it less capable of maintaining its structural integrity and resisting deformation.^22^ These findings may help explain the volumetric advantage observed in favor of G1. Additionally, degradation of the carboxymethylcellulose in diluted CaHA formulations could further impact the filler's long-term structural capacity.^23^

Conversely, the rationale behind using a product that simultaneously provides volume and collagen stimulation cannot be fully supported. Although biostimulatory effects are often cited as an advantage, volumetric outcomes are known to be compromised, and improvements in tissue quality are frequently demonstrated in noncomparative studies. Chang et al evaluated 25 patients injected with 3 mL of a mix (1.5 mL CaHA + 1.0 mL HA + 1.0 mL lidocaine) in the nasolabial fold. Qualitative analyses, which were not blinded, indicated improvement according to the authors; however, they did not present any statistical data. Similarly, Scardua et al demonstrated significant volumetric improvements in the frontal region using a mixture of CaHA and HA in a randomized, controlled, double-blind clinical trial involving 132 participants. Their results showed an increase in the Global Aesthetic Improvement Scale (GAIS) score at 180 days, highlighting the efficacy of the procedure. Nevertheless, this investigation lacked detailed quantitative analyses and used a different comparison model, contrasting CaHA alone with the mixture rather than using HA as the standard comparator.^4^

Alchemist-like approaches to creating newly mixed products may lead to the loss of certain original properties, which, when used separately, perform more effectively.^24^ Additionally, such product combinations (ie, HA + CaHA) are not recommended and explicitly contraindicated by manufacturers, increasing the risk of contamination and potentially leading to adverse events.^25^ Furthermore, unknown and unfavorable chemical interactions could occur.^26^ Although no serious adverse events occurred during the study, the limited sample size and short-term follow-up may not capture rare or delayed complications.

An alternative approach that may offer advantages is the application of different products in distinct anatomical planes rather than premixing them. In the study by Viscomi et al, the strategic placement of fillers, such as CaHA and HA, in separate planes preserved each material's unique rheological properties, maximizing their effectiveness. The results showed that the combination of supraperiosteal CaHA for structural support and biostimulation with subcutaneous HA for moldability and volumization produced significant aesthetic improvements in the lower third of the face, including the mandibular contour and chin projection. Additionally, this approach resulted in high patient satisfaction, as reflected in GAIS evaluations.^27^

As strengths, this study stands out for incorporating quantitative analyses, such as stereophotogrammetry, combined with validated qualitative tools, including the ATS, providing a comprehensive and objective evaluation of results. The direct comparison between isolated HA and a mixture of HA with CaHA addresses a relevant gap in the literature, particularly regarding the temporal region, an area often overlooked in research.

The study has some limitations, including a small sample size of 20 female participants, which may affect the generalizability of the finding, although 40 temporal areas were totally analyzed. Additionally, the 90-day follow-up period was intentionally selected to evaluate early volumetric outcomes rather than long-term neo-collagenesis effects. Another limitation of this study is the absence of patient-reported outcome measures, such as satisfaction surveys or quality of life assessments, which could provide additional insights into the perceived success of the treatments. Lastly, the study’s focus on the temporal region limits the applicability of the findings to other anatomical areas. Future studies with larger samples, split-face designs, evaluation in other facial areas, and longer follow-up periods are needed to further clarify the long-term efficacy and safety of different volumization strategies.

## CONCLUSIONS

In this study, the authors demonstrated that although both pure HA and HA–CaHA combination effectively reduced temporal hollowing, pure HA exhibited superior volumetric retention over time. The anticipated synergistic effect of the mixture did not translate into clinical advantages, suggesting that manual mixing may compromise material efficacy. Given the lack of manufacturer recommendations and the observed volumetric performance, pure HA remains the most predictable and evidence-supported choice for temporal volumization.
